# Substance Use Disorders (SUDs) and Risk of Cardiovascular Disease (CVD) and Cerebrovascular Disease (CeVD): Analysis of the Nationwide Inpatient Sample (NIS) Database

**DOI:** 10.7759/cureus.39331

**Published:** 2023-05-22

**Authors:** Harshil Patel, Urvish K Patel, Medhat Chowdhury, Andrew D Assaf, Chaithanya Avanthika, Mohammed A Nor, Mohamed Rage, Apoorva Madapu, Sravani Konatham, Mamatha Vodapally, Vatsalya Bhat, Anupa Gnawali, Mohamed Mohamed, Nawal Abdi, Faizan A Malik, Marcel Zughaib

**Affiliations:** 1 Cardiology, Ascension Providence Hospital, Southfield, USA; 2 Public Health and Neurology, Icahn School of Medicine at Mount Sinai, New York, USA; 3 Internal Medicine, Ascension Providence Hospital/MSUCHM, Southfield, USA; 4 Heart Institute, Ascension Providence Hospital, Southfield, USA; 5 Pediatrics, Icahn School of Medicine at Mount Sinai, Queens Hospital Center, New York City, USA; 6 Medicine and Surgery, Karnataka Institute of Medical Sciences, Hubli, IND; 7 Internal Medicine, Norman Bethune Health Science of Jilin University, Jilin, CHN; 8 Emergency Medicine, Northampton General Hospital, Northampton, GBR; 9 Internal Medicine, Huntsville Hospital, Huntsville, USA; 10 Internal Medicine, Kamineni Institute of Medical Sciences, Narketpalli, IND; 11 Internal Medicine, MNR Medical College, NTRUHS, Sangareddy, IND; 12 Internal Medicine, K. V. G. Medical College and Hospital, Sullia, IND; 13 Family Medicine, University of Cape Town, Caledon Provincial Hospital, Caledon, ZAF; 14 Internal Medicine, Wuhan University, Wuhan, CHN; 15 Internal Medicine, Capital Medical University, Beijing, CHN; 16 Internal Medicine, Texas Tech University Health Sciences Center at Permian Basin, Odessa, USA; 17 Cardiovascular Medicine, Ascension Providence Hospital, Southfield, USA

**Keywords:** smoking tobacco, amphetamine abuse, cocaine abuse, acute ischemic stroke, stroke, sudden cardiac death, marijuana use, marijuana, drug abuse, recreational substance abuse

## Abstract

Background: Substance use continues to be on the rise in the United States and has been linked to new onset cardiovascular diseases (CVDs) and cerebrovascular disorders (CeVDs). We aimed to study the association between the types of substance use disorders (SUDs) with specific subtypes of CVDs and CeVDs among hospitalized patients using the National Inpatient Sample (NIS) Database.

Methods: A retrospective study of the NIS database (2016-2017) using the ICD-10-CM codes was performed. The hospitalizations with a secondary diagnosis of SUDs were identified. Weighted univariate analysis using the Chi-square test and multivariate survey logistic regression analysis was performed to evaluate for the incidence, prevalence, and odds of association between vascular events and SUDs.

Results: There were a total of 58,259,589 hospitalizations, out of which 21.42% had SUDs. SUDs were more common in the younger age group of 18-50, males, and the lower median household income group. We found a significant association of acute ischemic stroke (AIS) with amphetamine dependence (adjusted odds ratio, aOR 1.23, 95% confidence interval, CI 1.14-1.33), cocaine-related disorders (1.17, 1.12-1.23), and nicotine dependence (1.42, 1.40-1.43). There was a significant association between intracerebral hemorrhage with amphetamine dependence (2.58, 2.26-2.93), cocaine-related disorders (1.62, 1.46-1.79), and alcohol-related disorders (1.35, 1.01-1.82). The association of subarachnoid hemorrhage (SAH) was noted to be higher with amphetamine dependence (1.82, 1.48-2.24) and nicotine dependence (1.47, 1.39-1.55). The patients with nicotine dependence had greater odds of having a myocardial infarction (1.85, 1.83-1.87), those with cocaine-related disorders had higher odds of having angina pectoris (2.21, 1.86-2.62), and patients with alcohol-related disorders had higher odds of developing atrial fibrillation (1.14, 1.11-1.17) in comparison to non-SUDs.

Conclusion: Our study demonstrates the variability of CVD and CeVD in patients hospitalized for SUD. Findings from our study may help promote increased awareness and early management of these events. Further studies are needed to evaluate the specific effects of frequency and dose on the incidence and prevalence of CVD and CeVD in patients with SUD.

## Introduction

The past few decades have seen a rise in the incidence and prevalence of cardiovascular disease (CVD) concomitant with substance use disorders (SUDs) among younger adults aged 18-45 years of age [1]. In the United States, common substances reportedly used include alcohol, tobacco, marijuana, sedatives, hallucinogens, cocaine, amphetamines, inhalants, and opioids [2].

The SUDs comprise an estimated 6.7% of all inpatient admissions and has been associated with greater costs and longer hospitalizations relative to non-SUD admissions [3]. A recent epidemiological study in British Columbia, Canada found SUD to be an independent risk factor associated with a greater prevalence and incidence of CVD on cross-sectional and longitudinal analysis respectively [4]. Accelerated atherosclerotic cardiovascular disease (ASCVD) is broadly suggested as a mechanism for increased CV events [5] in patients with SUD.

However, there is known variability in the types of CV events, depending on the type of agent used. For instance, an analysis of the National Health and Nutrition Examination Survey (NHANES) revealed a statistically significant association of non-fatal myocardial infarction with lifetime users of cocaine, when compared with non-users. The study did not reveal a significant association between ischemic stroke with cocaine users [6]. We, therefore, sought to investigate the spectrum of CVD and CeVD events in SUD among hospitalized patients in the United States.

## Materials and methods

Details of data

Data were obtained from the Agency for Healthcare Research and Quality's Healthcare Cost and Utilization Project (HCUP) Nationwide Inpatient Sample (NIS) files between January 2016 and December 2017. The NIS is the largest publicly available all-payer inpatient care database in the United States and contains discharge-level data provided by states that participate in the HCUP (including a total of 46 in 2011). This administrative dataset contains data on approximately 8 million hospitalizations in 1,000 hospitals that were chosen to approximate a 20% stratified sample of all US community hospitals, representing more than 95% of the national population. Criteria used for stratified sampling of hospitals into the NIS include hospital ownership, patient volume, teaching status, urban or rural location, and geographic region. Discharge weights are provided for each patient discharge record, which allows extrapolation to obtain national estimates. Each hospitalization is treated as an individual entry in the database and is coded with one principal diagnosis, up to 29 secondary diagnoses, and 15 procedural diagnoses associated with that stay. Detailed information on NIS is available at http://www.hcup-us.ahrq.gov/db/nation/nis/nisdde.jsp.

Demographic characteristics of the population

We performed a retrospective cross-sectional observational study on adult hospitalizations in the USA from 2016 to 2017. Secondary diagnoses of SUDs were identified using the ICD-10-CM code. Here are the listed ICD codes for SUDs that we used in our study, https://www.icd10data.com/ICD10CM/Codes/F01-F99/F10-F19. The SUDs include alcohol-related disorders, amphetamine dependence, cannabis-related disorders, cocaine-related disorders, hallucinogen-related disorders, inhalant-related disorders, opioid-related disorders, and nicotine dependence.

The comparison was made between US hospitalizations with a history of SUDs and hospitalizations without a history of SUDs. In each group, primary and secondary diagnoses of CVDs and CeVDs were identified. We considered angina pectoris, myocardial infarction (STEMI), sudden cardiac arrest, ischemic heart disease, and atrial fibrillation as CVDs and acute ischemic stroke (AIS), transient ischemic attack, and hemorrhagic stroke as CeVDs. ICD-10-CM codes for vascular diseases are described in Table 1. We used ICD-10-CM codes to identify comorbidities of hypertension, diabetes mellitus, obesity, dyslipidemia, renal failure, HIV/AIDS, solid tumors, depression, ischemic heart disease, atrial fibrillation, and congestive heart failure. Age <18 years and admissions with missing data for age, sex, and race were excluded. We obtained patient characteristics of interest (age, sex, race, insurance status, admission day, admission type, median household income category, and concomitant diagnoses) and hospital characteristics (hospital size, hospital region, and teaching versus nonteaching hospital). 

**Table 1:** Demographics of SUDs amongst USA hospitalizations. *No-SUDs defined by the hospitalizations/patients who are not having alcohol-related disorders, amphetamine dependence, cannabis-related disorders, cocaine-related disorders, hallucinogen-related disorders, inhalant-related disorders, opioid-related disorders, and nicotine dependence Column (%) comparisons were made between individual SUD vs. no-SUD SUD, substance use disorder

Variables	Alcohol-related disorders (n=3420548) (5.87%)	Amphetamine dependence (n=295060) (0.51%)	Cannabis-related disorders (n=1072879) (1.84%)	Cocaine-related disorders (n=341745) (0.59%)	Hallucinogen-related disorders (n=7345) (0.01%)	Inhalant-related disorders (n=2305) (0.0%)	Opioid-related disorders (n=889520) (1.53%)	Nicotine dependence (n=6451382) (11.07%)	No-SUDs* (n=45,778,806) (78.58%)	Total (n=58,259,589) (100%)	p value
Demographic and socioeconomic characteristics (%)											
Age groups (%)											< 0.0001>
Age group 18-50 years	42.61	76.17	75.17	57.87	82.91	67.25	52.86	35.72	31.83	34.4	
Age group 50-75 years	52.67	23.64	24.22	41.75	16.34	29.93	41.74	55.73	40.88	42.84	
Age group >75 years	4.73	0.19	0.62	0.38	0.75	2.82	5.41	8.55	27.29	22.27	
Sex											< 0.0001>
Male	71.81	59.02	57.54	59.05	62.42	60.09	44.67	49.84	38.14	42.10	
Female	28.19	40.98	42.46	40.95	37.58	39.91	55.33	50.16	61.86	57.90	
Race (%)											<0 .0001>
White	69.97	70.81	55.70	40.05	38.45	72.41	77.09	74.30	69.31	69.61	
Black	16.83	9.97	32.24	48.64	43.50	13.25	13.40	17.27	14.76	15.64	
Hispanic	10.70	14.96	10.07	10.34	16.49	10.82	7.98	6.46	12.11	11.30	
Asian or Pacific Islander	0.99	2.41	0.99	0.55	1.28	2.21	0.67	1.21	3.27	2.81	
Native American	1.51	1.85	1	0.42	0.28	1.32	0.86	0.75	0.54	0.64	
Median household income for patient's ZIP code (%)											< 0.0001>
0-25th percentile	34.77	39.52	42.33	52.87	46.09	40.68	35.33	38.80	29.12	31.04	
26th-50th percentile (median)	25.51	28.40	25.31	21.00	19.32	26.36	26.31	28.22	25.70	25.96	
51st to 75th percentile	22.28	21.00	19.71	15.61	18.76	18.86	22.24	20.70	24.08	23.43	
76th-100th percentile	17.43	11.08	12.65	10.51	15.83	14.09	16.12	12.28	21.10	19.57	
Primary payer insurance (%)											< 0.0001>
Medicare	28.96	14.64	18.82	22.11	14.66	25.81	36.38	41.29	51.04	47.49	
Medicaid	33.08	57.05	44.59	49.56	51.06	31.24	38.60	24.92	15.19	18.64	
Private	22.71	11.03	21.63	12.26	19.02	25.16	16.74	23.08	28.13	26.78	
Self	15.25	17.27	14.96	16.07	15.27	17.79	8.28	10.71	5.63	7.09	
Admission type (%)											< 0.0001>
Emergency and urgent	90.68	90.82	87.00	91.42	89.84	84.13	85.49	82.08	73.86	76.37	
Elective	9.32	9.18	13.00	8.58	10.16	15.87	14.51	17.92	26.14	23.63	
Admission day (%)											< 0.0001>
Weekdays (Monday-Friday)	75.71	75.14	77.44	74.62	75.43	79.18	76.92	78.58	80.15	79.54	
Weekends (Saturday-Sunday)	24.49	24.86	22.56	25.38	24.57	20.82	23.08	21.42	19.85	20.46	
Bedsize (%) (hospital size by number of beds)											< 0.0001>
Small	20.24	17.70	18.56	19.59	24.23	23.64	19.99	19.36	19.43	19.46	
Medium	29.75	24.71	28.43	30.28	28.25	34.27	28.11	28.96	29.51	29.40	
Large	50.02	57.59	53.01	50.14	47.52	42.08	51.90	51.69	51.06	51.14	
Location and teaching status (%)											< 0.0001>
Rural	7.95	9.31	7.30	4.05	4.08	11.26	7.70	11.31	8.95	9.08	
Urban, non-teaching	24.39	28.34	21.78	19.06	20.63	17.79	26.35	25.19	24.926	24.86	
Urban, teaching	67.66	62.35	70.90	76.89	75.29	70.93	65.95	63.50	66.13	66.06	
Hospital region (%)											< 0.0001>
Northeast	21.91	3.32	18.86	27.58	31.93	18.66	22.35	16.28	19.52	19.30	
Midwest or North Central	21.95	14.46	24.12	20.79	18.58	18.87	20.27	25.45	20.97	21.54	
South	35.85	28.13	35.35	42.63	36.15	49.46	34.92	42.74	40.23	40.14	
West	20.30	54.09	21.67	9.01	13.34	13.02	22.26	14.53	19.28	19.03	
Concurrent comorbidities (%)											
Diabetes (%)	16.20	16.10	15.30	21.58	12.73	15.40	21.38	27.42	27.65	26.54	< 0.0001>
Hypertension (%)	51.86	30.81	31.96	47.29	28.25	33.62	43.87	57.10	56.29	55.29	< 0.0001>
Obesity (%)	8.88	8.36	11.27	9.79	8.51	9.76	13.75	15.95	15.88	15.28	< 0.0001>
Dyslipidemia (%)	19.90	10.12	14.16	16.84	8.85	16.27	18.34	33.22	33.65	31.98	< .0001>
Renal failure (%)	17.19	16.65	13.74	24.06	15.52	14.75	20.40	20.18	25. 22	23.85	< .0001>
Smoking (%)	46.54	57.52	49.39	56.77	41.87	41.00	42.92	100.00	0.00	16.00	< 0.0001>
AIDS (%)	100.00	1.04	0.76	2.27	0.68	1.30	0.69	0.41	0.17	6.09	< 0.0001>
Metabolic syndrome (%)	0.09	0.08	0.11	0.07	0.07	0.00	0.13	0.17	0.18	0.17	< 0.0001>

Outcomes

The primary aim of this study was to evaluate the incidence and prevalence of CVDs and CeVDs amongst patients with SUDs. The secondary aim of this study was to evaluate the odds of CVDs and CeVDs amongst SUDs.

Statistical analysis

We analyzed the data using SAS software (Version 9.4) (SAS, Cary, NC). We performed univariate analysis to find the association of SUDs with CVDs and CeVDs. Categorical variables were evaluated using Chi-square and a *t*-test was used for continuous variables. Multivariable survey logistic regression models were generated to predict the association of CVDs and SUDs as well as CeVDs and SUDs after adjusting for confounding variables. There was no pre-decided sample size calculation. The p-value <0.05 was considered significant and OR with its 95% CI was calculated.

Details of confounders

In regression analysis, the models were adjusted with socio-demographic variables like age, race, ethnicity, annual household income, education status, and comorbidities like diabetes, cholesterol, hypertension cardiovascular health, obesity, and preventive aspirin use.

## Results

Disease hospitalizations

We found 58,259,589 hospitalizations [weighted after removing missing data for age, sex, and race (unweighted:11,651,925)] from January 2016 to December 2017 after excluding patients with ages <18 years. Out of these hospitalizations, 21.42% had a history (secondary diagnosis) of major SUDs. The prevalence of alcohol-related disorders was 5.87%, amphetamine dependence was 0.51%, cannabis-related disorders was 1.84%, cocaine-related disorders were 0.59%, hallucinogen-related disorders were 0.01%, opioid-related disorders were 1.53%, and nicotine dependence was 11.07%.

Demographic characteristics of patients with a history of substance abuse

Among patients admitted to the hospital with a diagnosis of SUD, the mean age was found to be relatively lower for all the subtypes of SUDs: alcohol-related disorders (52 years old, standard error - SE: 0.02), amphetamine dependence (40 years old, SE: 0.05), cannabis-related disorder (38 years old, SE: 0.03), cocaine-related disorders (46 years old, SE: 0.05), hallucinogen-related disorders (36 years old, SE: 0.33), inhalant-related disorders (43 years old, SE: 0.71), opioid-related disorders (48 years old, SE: 0.04), and nicotine dependence (54 years old, SE: 0.01) in comparison to patients without a diagnosis of SUD (59 years old, SE: 0.01) (p < 0.0001). The patients were divided into three age groups for subgroup analysis: 18-50, 50-75, and >75 years old. All SUDs were more prevalent in the age group of 18-50 in comparison to the other groups. Males had a higher prevalence of all types of SUDs in comparison to females (p < 0.0001). Among all hospitalized patients, Native Americans were found to have a significantly higher prevalence of alcohol-related disorders, amphetamine dependence, inhalant-related disorders, opioid-related disorders, and nicotine dependence in comparison to other ethnic groups (p < 0.0001). Similarly, African Americans had a significantly higher prevalence of cannabis-related disorders, cocaine-related disorders, and hallucinogen-related disorders (p < 0.0001). We performed a subgroup analysis to look for differences in the prevalence of SUDs among hospitalized patients belonging to different economic backgrounds. The patients with the lowest median household income (0-25th percentile) had a higher prevalence of all types of SUDs in comparison to other household income groups (p < 0.0001). The admitted patients who were self-payers had a significantly higher prevalence of alcohol use disorders and nicotine dependence as compared to all other insurance groups (Private, Medicare, and Medicaid). The rest of the SUDs was more prevalent among the patients insured with Medicaid (p < 0.0001). Among all hospitalized patients with a diagnosis of SUD, a significantly higher proportion of patients were admitted over the weekend (p < 0.0001) (Table 1).

Incidence and prevalence of cerebrovascular disorders

The incidence of AIS was higher in patients with cocaine-related disorders (1.71%), and nicotine dependence (2.28%) when compared to those without SUDs (1.70%) (p < 0.0001). The incidence of intracerebral hemorrhage (ICeH) was higher in patients with amphetamine dependence (0.40%), and cocaine-related disorders (0.35%) in comparison to those without SUDs (0.29%) (p < 0.0001). The incidence of subarachnoid hemorrhage (SAH) was higher in patients admitted with alcohol-related disorders (0.27%), amphetamine dependence (0.40%), and cocaine-related disorders (0.35%) when compared to those with no SUDs (0.24%) (p < 0.0001). The prevalence of a history of AIS was higher in patients admitted with cocaine-related disorders (2.22%) in comparison to those with no SUDs (2.14%) (p < 0.0001). There was a higher prevalence of history of ICeH in patients admitted with alcohol-related disorders (0.42%), amphetamine dependence (0.60%), and cocaine-related disorders (0.53%) in comparison to those patients with no SUDs (0.33%) (p < 0.0001). The prevalence of a history of SAH was higher among patients with amphetamine dependence (0.23%), cocaine-related disorders (0.23%), and nicotine dependence (0.18%) in comparison to those without SUDs (0.14%) (p < 0.0001) (Table 2).

**Table 2:** Incidence and prevalence of CeVDs and CVDs amongst active SUDs patients. ≤10 (-) indicated unidentified lower frequencies Column (%) comparisons were made between Individual SUD vs. no-SUD CeVDs, cerebrovascular disorders; SUD, substance use disorder; AIS, acute ischemic stroke; TIA, transient ischemic attack; ICeH, intracerebral hemorrhage; SAH, subarachnoid hemorrhage; CVDs, cardiovascular disorders; SCA, sudden cardiac arrest; IHD, ischemic heart disease; MI, myocardial infarction; Afib, atrial fibrillation

Outcomes	Alcohol-related disorders (n=3420548) (5.87%)	Amphetamine dependence (n=295060) (0.51%)	Cannabis-related disorders (n=1072879) (1.84%)	Cocaine-related disorders (n=341745) (0.59%)	Hallucinogen-related disorders (n=7345) (0.01%)	Inhalant-related disorders (n=2305) (0.0%)	Opioid-related disorders (n=889520) (1.53%)	Nicotine dependence (n=6451382) (11.07%)	No-SUDs* (n=45,778,806) (78.58%)	Total (n=58,259,589) (100%)	p value
Incidence (new primary diagnosis) of CeVDs (%)											
AIS (%)	1.29	1.13	1.12	1.71	0.48	≤10 (-)	0.54	2.28	1.70	1.71	<0.0001
TIA (%)	0.18	0.09	0.19	0.18	≤10 (-)	≤10 (-)	0.13	0.37	0.41	0.38	<0.0001
ICeH (%)	0.2	0.40	0.13	0.35	≤10 (-)	≤10 (-)	0.07	0.18	0.29	0.23	<0.0001
SAH (%)	0.27	0.40	0.13	0.35	≤10 (-)	≤10 (-)	0.07	0.18	0.24	0.23	<0.0001
Prevalence (primary and secondary diagnosis) of CeVDs (%)											
AIS (%)	1.69	1.64	1.35	2.22	0.95	0.87	0.95	0.69	2.14	2.14	<0.0001
TIA (%)	0.29	0.16	0.25	0.28	≤10 (-)	≤10 (-)	0.23	0.56	0.67	0.57	<0.0001
ICeH (%)	0.42	0.60	0.22	0.53	≤10 (-)	≤10 (-)	0.14	0.16	0.33	0.40	<0.0001
SAH (%)	0.13	0.23	0.13	0.23	≤10 (-)	≤10 (-)	0.9	0.18	0.14	0.14	<0.0001
Incidence (new primary diagnosis) of CVDs (%)											
SCA (%)	0.06	0.04	0.03	0.09	≤10 (-)	≤10 (-)	0.04	0.04	0.05	0.05	<0.0001
IHD (%)	1.81	1.75	2.28	2.97	1.09	0.65	1.24	5.74	3.32	3.44	<0.0001
MI (%)	1.21	1.38	1.62	2.01	1.02	≤10 (-)	0.78	3.97	2.01	2.15	<0.0001
Angina pectoris (%)	0.04	0.04	0.05	0.14	≤10 (-)	≤10 (-)	0.03	0.08	0.06	0.06	<0.0001
Afib (%)	0.99	0.35	0.39	0.41	0.20	≤10 (-)	0.31	0.85	1.18	1.10	<0.0001
Prevalence (primary and secondary diagnosis) of CVDs (%)											
SCA (%)	0.79	0.77	0.42	0.95	0.68	≤10 (-)	0.75	0.63	0.67	0.67	<0.0001
IHD (%)	13.41	9.80	9.87	16.60	5.85	10.85	14.10	25.03	22.84	22.05	<0.0001
MI (%)	2.18	2.57	2.30	3.60	1.70	1.30	1.85	5.31	3.33	3.44	<0.0001
Angina pectoris (%)	0.16	0.14	0.18	0.54	0.34	≤10 (-)	0.14	0.25	0.19	0.19	<0.0001
Afib (%)	7.03	2.77	2.77	3.94	1.57	3.47	5.41	7.65	12.21	11.03	<0.0001

Incidence and prevalence of cardiovascular disorders

The incidence of angina pectoris was higher in patients with cocaine-related disorders (0.14%) and nicotine dependence (0.08%) in comparison to those without SUDs (0.06%) (p < 0.0001). Similarly, the prevalence of angina pectoris was higher in patients with cocaine-related disorders (0.54%), and nicotine dependence (0.25%) in comparison to those with no SUDs (0.19%) (p < 0.0001). The prevalence of a history of myocardial infarction (MI) was higher in patients with cocaine-related disorders (3.60%) and nicotine dependence (5.31%) in comparison to those with no SUDs (3.33%) (p < 0.0001). The incidence (1.71% vs. 1.70%) and prevalence (2.22% vs. 2.14%) of AIS were higher amongst patients with cocaine-related disorders compared to those without SUDs.

Multivariable regression showing odds of hospitalizations with new onset of cerebrovascular disorders and cardiovascular amongst SUDs patients

The odds of having hospitalizations with new onset of AIS were higher in patients with amphetamine dependence (OR 1.23, 95%CI 1.14-1.33; p < 0.0001), cocaine-related disorders (1.17, 1.12-1.23; p < 0.0001), and nicotine dependence (1.42, 1.40-1.43; p < 0.0001). The odds of having hospitalizations with new onset ICH were higher in patients with amphetamine dependence (2.58, 2.26-2.93; p < 0.0001), cocaine-related disorders (1.62, 1.46-1.79; p < 0.0001), and alcohol-related disorders (1.35, 1.01-1.82; p=0.0439). The odds of having hospitalizations with new onset SAH were higher in patients with amphetamine dependence (1.82, 1.48-2.24; p < 0.0001), and nicotine dependence (1.47, 1.39-1.55; p < 0.0001). The odds of having hospitalizations with new-onset myocardial infarction were higher in patients with nicotine dependence (1.85, 1.83-1.87; p < 0.0001) and cocaine-related disorders (1.09, 1.04-1.14; p=0.0002). The odds of having hospitalizations with new-onset angina pectoris were higher in patients with cocaine-related disorders (2.21, 1.86-2.62; p < 0.0001). The odds of having hospitalizations with new-onset atrial fibrillation were higher in patients with alcohol-related disorders (1.14, 1.11-1.17; p < 0.0001) (Table 3).

**Table 3:** Risk of having hospitalizations with new onset of CeVD and CVD amongst active SUDs patients. CeVD, cerebrovascular disorder; CVD, cardiovascular disease; SUD, substance use disorder; AIS, acute ischemic stroke; TIA, transient ischemic attack; ICeH, intracerebral hemorrhage; SAH, subarachnoid hemorrhage; SCA, sudden cardiac arrest; MI, myocardial infarction; AFib, atrial fibrillation; aOR, adjusted odds ratio; CI, confidence inrterval

Outcomes	Alcohol-related disorder aOR (95%CI) p-value	Amphetamine dependence aOR (95%CI) p-value	Cannabis-related disorders aOR (95%CI) p-value	Cocaine-related disorder aOR (95%CI) p-value	Hallucinogen-related disorder aOR (95%CI) p-value	Inhalant-related disorder aOR (95%CI) p-value	Opioid-related disorder aOR (95%CI) p-value	Nicotine dependence aOR (95%CI) p-value	c value
Model 1 AIS	0.68 (0.61-0.76) <.0001	1.23 (1.14-1.33) <.0001	1.03 (0.99-1.06) 0.2058	1.17 (1.12-1.23) <.0001	0.43 (0.28-0.67) 0.0002	0.39 (0.14-1.05) 0.0619	0.38 (0.36-0.40) <.0001	1.42 (1.40-1.43) <.0001	0.783
Model 2 TIA	0.72 (0.54-0.96) <.0001	0.64 (0.50-0.82) 0.0233	0.86 (0.78-0.95) 0.0032	0.77 (0.67-0.89) 0.0002	0.59 (0.22-1.58) 0.2967	0.56 (0.08-3.97) 0.5596	0.43 (0.38-0.49) <.0001	0.95 (0.92-0.98) 0.0003	0.778
Model 3 ICeH	1.35 (1.01-1.82) 0.0439	2.58 (2.26-2.93) <.0001	0.78 (0.71-0.87) <.0001	1.62 (1.46-1.79) <.0001	0.34 (0.11-1.07) 0.0650	-	0.37 (0.32-0.43) <.0001	0.76 (0.73-0.79) <.0001	0.771
Model 4 SAH	0.82 (0.50-1.35) 0.4345	1.82 (1.48-2.24) <.0001	0.94 (0.82-1.08) 0.3917	1.09 (0.91-1.31) 0.3551	0.52 (0.13-2.08) 0.3524	-	0.38 (0.31-0.47) <.0001	1.47 (1.39-1.55) <.0001	0.695
Model 5 SCA	1.23 (0.73-2.05) 0.4404	1.01 (0.68-1.48) 0.9731	0.95 (0.78-1.17) 0.6571	1.24 (0.99-1.55) 0.0581	1.91 (0.71-5.13) 0.1983	2.45 (0.34-17.4) 0.3723	1.20 (1.00-1.43) 0.0460	0.85 (0.78-0.92) 0.0001	0.544
Model 7 MI	0.47 (0.43-0.52) <.0001	0.90 (0.84-0.96) 0.0013	0.96 (0.93-0.99) 0.0124	1.09 (1.04-1.14) 0.0002	0.64 (0.46-0.90) 0.0090	0.40 (0.18-0.90) 0.0266	0.37 (0.36-0.39) <.0001	1.85 (1.83-1.87) <.0001	0.785
Model 8 Angina pectoris	0.49 (0.29-0.84) 0.0095	0.76 (0.50-1.14) 0.1803	0.74 (0.61.0.89) 0.0013	2.21 (1.86-2.62) <.0001	0.70 (0.18-2.79) 0.6147	-	0.46 (0.36-0.58) <.0001	1.08 (1.01-1.15) 0.0312	0.598
Model 9 AFib	1.14 (1.11-1.17) <.0001	0.97 (0.86-1.09) 0.5864	0.90 (0.84-0.95) 0.0002	0.93 (0.86-1.01) 0.0987	0.56 (0.29-1.06) 0.0758	0.77 (0.29-2.04) 0.5921	0.39 (0.36-0.41) <.0001	0.78 (0.76-0.79) <.0001	0.711

## Discussion

Our analysis of 58,259,589 hospitalizations revealed the following findings: (1) amphetamine and cocaine use were associated with greater odds of ischemic stroke and ICH, (2) cocaine but not amphetamine use was associated with increased odds of angina and acute MI, (3) alcohol use was associated with an increased odds of ICH and atrial arrhythmias, (4) nicotine use was associated with an increased odds of ischemic stroke, acute MI, and sudden cardiac arrest (SCA).

Both amphetamine and cocaine are vasoactive agents that exert their effect through catecholaminergic modulation and have been previously associated with both ischemic and hemorrhagic stroke [7]. A retrospective study in young adults by Westover et al. noted a statistically significant association between cocaine use and both ischemic and hemorrhagic stroke [8]. Amphetamine use was only associated with hemorrhagic strokes and not ischemic strokes [8]. In addition, a review of cases reported in the literature also found a preponderance of amphetamines for hemorrhagic stroke [9]. Our analysis echoed these trends, whereby a stronger association was demonstrated between amphetamine use and hemorrhagic stroke (2.58, 2.26-2.93) as compared to ischemic stroke (1.23, 1.14-1.33), albeit both associations were statistically significant. Compared to the study by Westover et al, the statistical significance of the latter is likely due to the larger sample size, considering the lower event rate of ischemic strokes demonstrated in prior studies. Mechanistically, cocaine and amphetamines can cause hemorrhagic stroke through acute hypertension, aneurysm formation, and rupture. For ischemic strokes, common mechanisms include arrhythmias with cardio-embolism, severe vasospasm, and accelerated atherosclerosis [7]. Cocaine additionally may trigger platelet hyper-aggregation and arterial dissections as additional mechanisms for ischemic stroke [7]. We also found a statistically significant association between amphetamine and SAH, which lends support to a recent retrospective study by Noblett et al. that indicated an increased risk of rupture and SAH at smaller aneurysmal size in patients with a history of methamphetamine use [10].

In terms of involvement of the cardiovascular system, hospitalization with angina pectoris and acute MI was greater in patients with cocaine use (1.09, 1.04-1.14; p=0.0002) but not amphetamine use. This is contrasted to the findings by Westover et al. who found a statistically significant association of acute MI with both cocaine and amphetamine use among hospitalizations in Texas [11]. Their study also demonstrated regional variation in the strength of association between amphetamine use and acute MI, suggesting areas with heavier use might be more susceptible to cardiovascular events. Further studies investigating this association in other regions and the frequency/amount of amphetamine use with incident events will lend more clarity to the discrepancy observed between our findings and that of Westover et al.

The increased legalization of marijuana and its rising prevalence has led to increased concern regarding its long-term effects on the cardiovascular system (World Drug Report 2019, United Nations) [12]. Our study revealed that while there was a statistically significant increase in both the incidence and prevalence of ischemic heart disease and MI in hospitalized patients with cannabis use, no significant association was noted when adjusted for potential confounders. Our results are consistent with data from the Behavioral Risk Factor Surveillance System (BRFSS) by Jivanji et al. (2020) [13] that also failed to reveal an association between cardiovascular disease and cannabis use. Furthermore, longitudinal studies investigating marijuana use and the development of cardiovascular disease in 18-30-year-olds also showed no change in the risk of developing cardiovascular disease later in life [14].

We found significantly increased odds of hospitalization with ICH in patients with alcohol-related disorders (1.35, 1.01-1.82; p=0.0439). A prior retrospective study by Casolla et al. demonstrated similar findings with the proposed mechanism being through coagulopathy and platelet dysfunction [15]. In keeping with previous studies, we also demonstrated a statistically significant association between atrial arrhythmias and alcohol use [16]. This is proposed to occur through alcohol-mediated myocyte injury and autonomic dysfunction leading to an increased likelihood for the formation of atrial re-entry circuits, precipitating atrial fibrillation [17]. Chelikam et al., in the NHANES study, evaluated 263465 respondents with CVDs, in which marijuana (1.98, 1.98-1.98), injectable illegal drug use (2.15, 2.14-2.15), and cigarette smoking (1.55, 1.55-1.55) had significantly increased the risk CVDs [18].

Tobacco use is a well-established risk factor and has been shown to have a dose-dependent effect on the risk of ischemic stroke, MI, and ruptured cerebral aneurysms [19-21]. Our findings are in agreement with prior studies; nicotine use was independently associated with ischemic stroke (1.23, 1.14-1.33; p < 0.0001), MI (1.09, 1.04-1.14; p=0.0002), and SAH (1.47, 1.39-1.55; p < 0.0001). Nicotine causes an increased adrenergic drive, combined with the toxic and prothrombotic effects of oxidant chemicals and carbon monoxide leading to ischemic events [22]. The increased shear stress and endothelial inflammation are also suspected to be the mechanisms behind aneurysm formation and rupture in aneurysmal SAH [21].

Strengths and limitations

Our study showed that there is a higher risk of CVDs among patients with SUDs, even though the evidence described in our findings is based on administrative billing codes, producing a decreased overall sensitivity when compared with clinical diagnosis. The data we provided, together with the relevant literature, show the importance of better education about drug use problems and the effects they have on cardiovascular health. While we were able to demonstrate an association of different types of SUDs with different subtypes of CVDs and CeVDs, our study was limited in further delineating the specific doses or frequency of drug uses and their association with CVDs and CeVDs. Moreover, the study was designed to establish association and not causality.

## Conclusions

In conclusion, our study highlights the significant variability among CVD and CeVD events among patients hospitalized for SUD. Our results may aid in raising the index of suspicion and early management, especially in young adults hospitalized for SUD. Further studies are needed to evaluate other parameters such as the frequency and dose-relationship of different substances and their effect on CVD and CeVD in hospitalized patients.
